# Supplementary material for: Synthesis of Water-Dispersed Sulfobetaine Methacrylate–Iron Oxide Nanoparticle-Coated Graphene Composite by Free Radical Polymerization
Source: Polymers (Basel). 2022 Sep 17;14(18):3885. doi: 10.3390/polym14183885 (PMC9505676; doi:10.3390/polym14183885)
Supplement: Supplementary file 1 [file polymers-14-03885-s001.zip › polymers-1891700-supplementary.pdf]

Supplementary Materials

Synthesis of Water-Dispersed Sulfobetaine Methacrylate-Iron Oxide Nanoparticles Coated Graphene Composite by Free Radical Polymerization

Suguna Perumal [1,2,*,†], Raji Atchudan [2,3,†], Yong Rok Lee [2,*]

[1] *Department of Chemistry, Sejong University, Seoul 143-747, Republic of Korea*

[2] *School of Chemical Engineering, Yeungnam University, Gyeongsan, Gyeongsangbuk-do 38541, Republic of Korea.*

[3] *Department of Chemistry, Saveetha School of Engineering, Saveetha Institute of Medical and Technical Sciences, Chennai-602105, Tamil Nadu, India*

* Correspondence: suguna.perumal@gmail.com (S.P.); yrlee@yu.ac.kr (Y.R.L.)

[†] These authors contributed equally to this work

Poly[2-(Methacryloyloxy)ethyl]dimethyl-(3-sulfopropyl)ammonium hydroxide] composite

The preparation of poly[2-(Methacryloyloxy)ethyl]dimethyl-(3-sulfopropyl)ammonium hydroxide] composite (PSB) was prepared as shown in **Scheme 1**. Monomer SB (500 mg, 1.78 mmol) and ACVA (25.0 mg, 0.089 mmol) in 70 mL of DI water were heated at 70 °C for 6 h in a sonication bath. Then the composite PSB was purified by dialysis in DI water using 3K cellulose membrane for 3 days, the water was replaced twice a day. After dialysis, the solution was dried in a freeze dryer that yielded white powder of PSB. The prepared PSB was characterized using size-exclusion chromatography (SEC) (Waters, Alliance e2695 with Waters 2414 Refractive index detector) and thermogravimetric analysis. The molecular number of PSB was measured as 24536 g/mol with PD value as 1.8 using SEC.

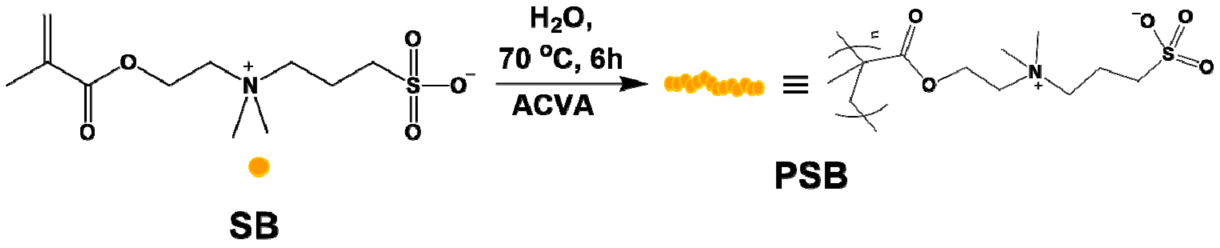

Scheme 1. Synthesis of poly[2-(Methacryloyloxy)ethyl]dimethyl-(3-sulfopropyl)ammonium hydroxide].

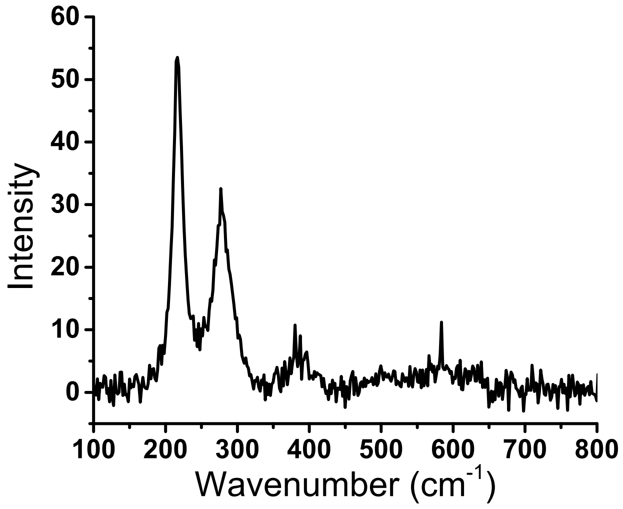

Figure S1. Raman spectrum of IONPs.

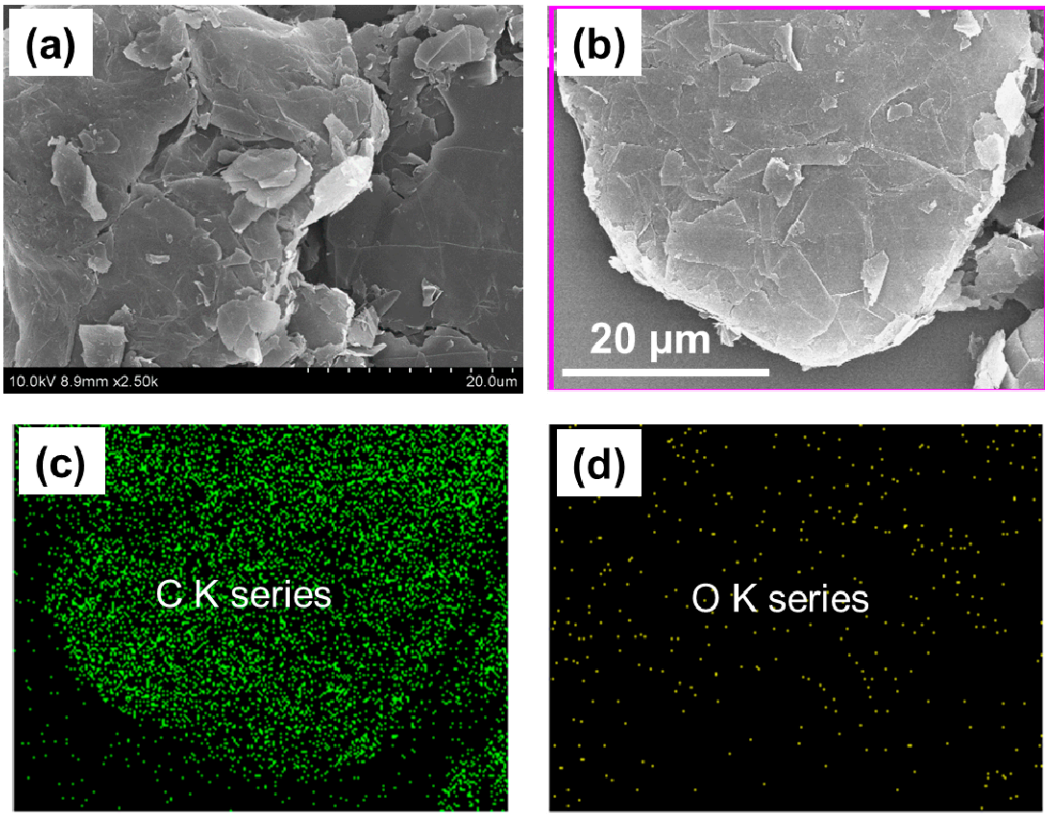

Figure S2. FESEM images of G (a and b). Elemental image of G (c) C K series, (d) O K series.

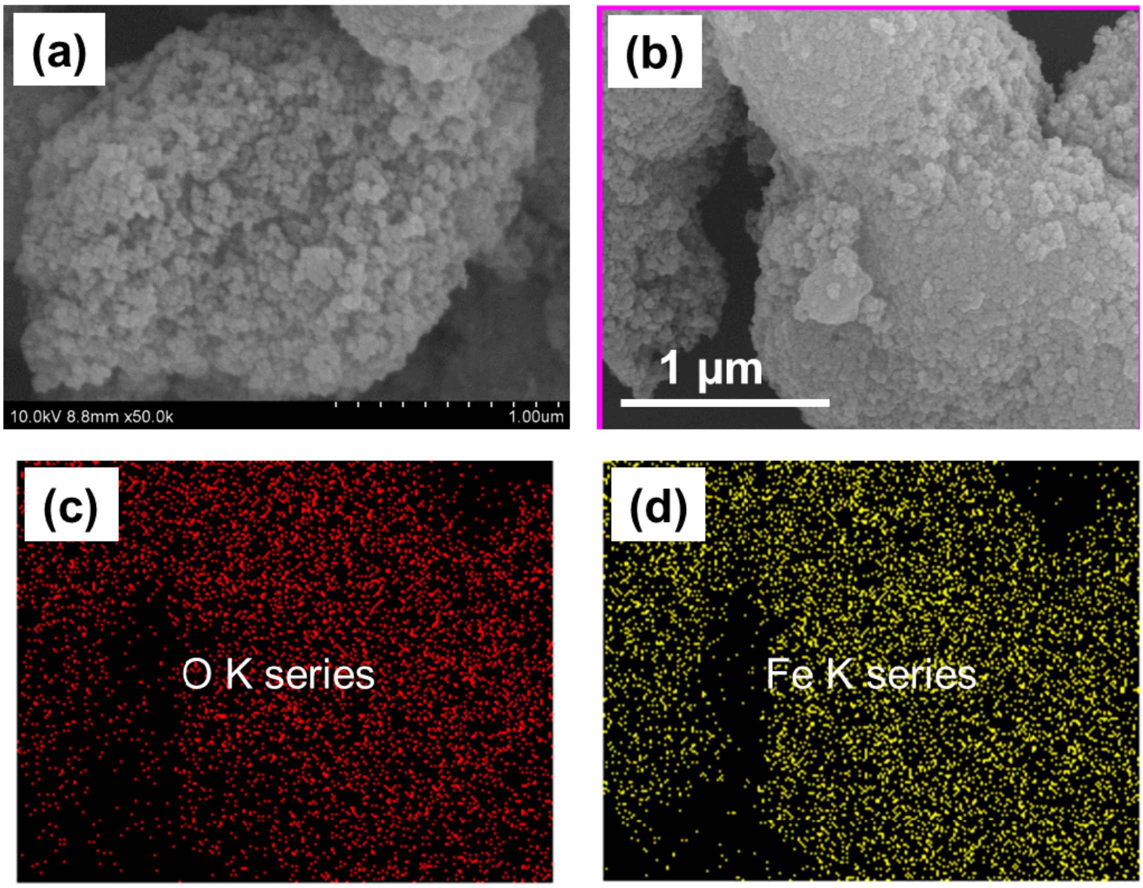

Figure S3. FESEM images of IONPs (a and b) and their elemental image (c) O K series, (d) Fe K series.

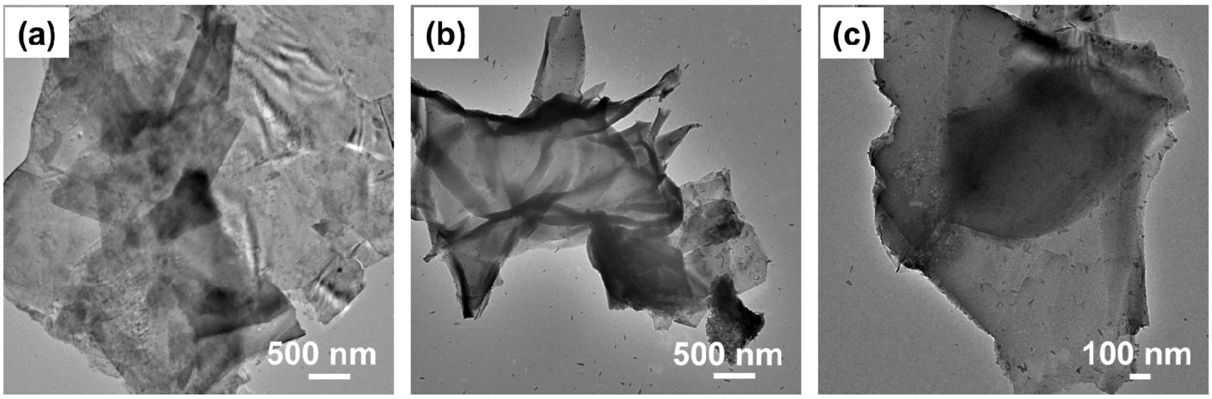

Figure S4. TEM images of G with different magnifications.

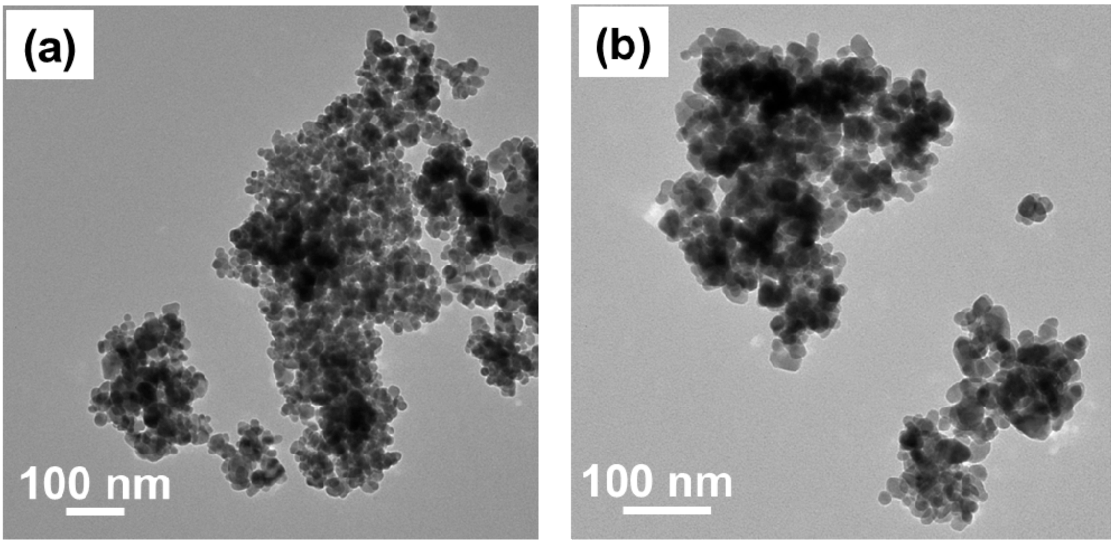

Figure S5. TEM images of IONPs (a and b).

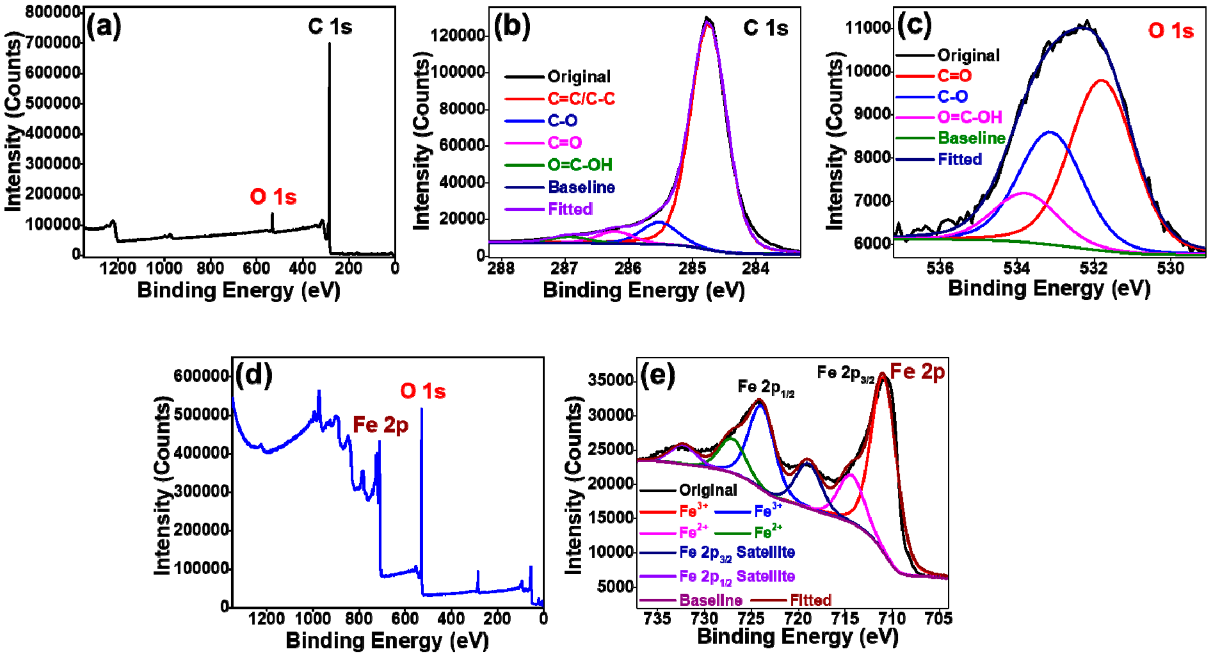

Figure S6. XPS spectra of G (a) survey spectra, (b) C 1s, and (c) O 1s. XPS spectra of IONPs (d) survey spectrum and (e) Fe 2p.
